# Two-component vaccine consisting of virus-like particles displaying hepatitis C virus envelope protein 2 oligomers

**DOI:** 10.1038/s41541-022-00570-1

**Published:** 2022-11-15

**Authors:** Jannick Prentoe, Christoph M. Janitzek, Rodrigo Velázquez-Moctezuma, Andreas Soerensen, Thomas Jørgensen, Stine Clemmensen, Vladislav Soroka, Susan Thrane, Thor Theander, Morten A. Nielsen, Ali Salanti, Jens Bukh, Adam F. Sander

**Affiliations:** 1 Copenhagen Hepatitis C Program (CO-HEP), Department of Infectious Diseases, Copenhagen University Hospital, Hvidovre and Department of Immunology and Microbiology, University of Copenhagen, Copenhagen, Denmark; 2 Centre for Medical Parasitology at the Department of Immunology and Microbiology, University of Copenhagen, and Department of Infectious Diseases, Rigshospitalet, Copenhagen University Hospital, Copenhagen, Denmark; 3 ExpreS2ion Biotechnologies, SCION-DTU Science Park, Hørsholm, Denmark; 4 AdaptVac Aps, Hørsholm, Denmark

**Keywords:** Hepatitis C, Conjugate vaccines, Hepatitis C virus

## Abstract

Development of B-cell-based hepatitis C virus (HCV) vaccines that induce broadly neutralizing antibodies (bNAbs) is hindered by extensive sequence diversity and low immunogenicity of envelope glycoprotein vaccine candidates, most notably soluble E2 (sE2). To overcome this, we employed two-component approaches using self-assembling virus-like particles (cVLPs; component 1), displaying monomeric or oligomeric forms of HCV sE2 (sE2_mono_ or sE2_oligo_; component 2). Immunization studies were performed in BALB/c mice and the neutralizing capacity of vaccine-induced antibodies was tested in cultured-virus-neutralizations, using HCV of genotypes 1–6. sE2-cVLP vaccines induced significantly higher levels of NAbs (*p* = 0.0065) compared to corresponding sE2 vaccines. Additionally, sE2_oligo_-cVLP was superior to sE2_mono_-cVLP in inducing bNAbs. Interestingly, human monoclonal antibody AR2A had reduced binding in ELISA to sE2_oligo_-cVLP compared with sE2_mono_-cVLP and competition ELISA using mouse sera from vaccinated animals indicated that sE2_oligo_-cVLP induced significantly less non-bNAbs AR2A (*p* = 0.0043) and AR1B (*p* = 0.017). Thus, cVLP-displayed oligomeric sE2 shows promise as an HCV vaccine candidate.

## Introduction

Hepatitis C virus (HCV) is a highly diverse virus with six clinically relevant genotypes and large variation in geographic distribution^1^. Globally, more than 1 million people become chronically infected every year and at least 58 million people suffer from chronic HCV infection^2^, increasing their risk of end-stage liver diseases, such as cirrhosis and cancer. An estimated 290.000 people die every year from chronic-HCV-related disease and while effective drugs targeting virus functions are now available, the disease burden is still increasing globally due to frequent undiagnosed infection, high drug cost, and poor healthcare infrastructure in developing countries^3^. Due to these challenges, the World Health Organization (WHO) explicitly urges research into developing HCV vaccines that prevents chronicity^4^ and this need was recently further emphasized by internationally renowned HCV experts^5^ and by the American Association for the Study of Liver Diseases (AASLD)^6^.

A lot has been learned from studying HCV clearance and potential vaccine candidates in animal models, most notably in chimpanzees^7^. While such studies offer important clues to inform vaccine development they do not guarantee final vaccine efficacy in humans^8,9^. This was recently emphasized for a T-cell based HCV vaccine approach, using recombinant chimpanzee adenovirus 3 vector priming followed by a recombinant modified vaccinia Ankara boost, which apparently protected chimpanzees from developing chronicity^10^, but was recently found in a phase I/II clinical T-cell based HCV vaccine trial, to successfully induce anti-HCV T-cell responses that lowered peak viremia, but without reducing HCV chronicity^11^. It has become apparent that neutralizing antibodies (NAbs) targeting the HCV envelope proteins E1, and especially E2, are critical in preventing chronicity^12,13^. Additionally, it was recently found that cross-genotype-reactive broadly NAbs (bNAbs) were associated with viral clearance^14^. While efforts to use E1/E2 complexes as vaccine antigens are ongoing^15–17^, most antibody-based vaccine research has focused on transmembrane truncated, soluble, E2 (sE2), which recapitulates many important bNAb epitopes^18,19^. The relevance of basing HCV vaccines on E2 was further supported by our recent finding that it was challenging for HCV to evade antibodies targeting the E2 antigenic region 3 (AR3)^20^. Another E2 epitope of vaccine interest is the linear E2 epitope termed, antigenic site 412 (AS412), which, in the absence of interfering antibodies against another part of E2^21^, also shows potential as a vaccine target. In addition, it was recently found that AS412 could induce NAbs when stabilized either on hepatitis B surface antigen or displayed as a peptide mimic on anti-idiotypic antibodies^22,23^. However, an inherent problem is the induction of antibodies against poorly conserved epitopes, such as antigenic region 1 (AR1)^24^, as well as against epitopes that are highly cryptic in the context of virus particles, such as antigenic region 2 (AR2)^25^.

While many research groups have contributed ways to improve immunogenicity of sE2^26–31^, it remains challenging to produce HCV vaccine antigens that are both immunogenic and adaptable to proven vaccine modalities. In the attempt to overcome this barrier, sE2 has been genetically fused to different proteins, which can self-assemble into nanoparticles (NP) displaying multiple copies of monomeric sE2^30,32,33^. This offers a way to boost the immunogenicity of sE2, by allowing the antigen to be delivered in a multivalent and highly repetitive format^34^. However, it is currently unclear whether bNAb epitope display is best achieved using monomeric or oligomeric forms of sE2^35^. Indeed, aggregate forms of sE2 have been shown to induce higher levels of bNAbs^29^, which could reflect that bNAb epitopes are relatively more abundant or immuno-dominant in multimeric sE2 assemblies compared to monomeric sE2. However, this could also simply be a result of larger multimers generally being more immunogenic, due to increased uptake by professional antigen presenting cells and increased avidity of B-cell receptor binding. Comparing monomeric and oligomeric sE2 as vaccine antigens could be performed using a two-component vaccine technology in which both monomeric and multimeric sE2 could be delivered by the same highly immunogenic virus-like particle (VLP) platform. To this end, the SpyTag/SpyCatcher Acetinobacter phage AP205 capsid virus-like particle platform (cVLP)^36^ enables unidirectional, high-density display of both simple (e.g., monomeric) and complex (e.g., multimeric) antigens on the surface of pre-assembled cVLPs. Also, this vaccine platform has recently been shown to be highly immunogenic and safe in animals^37^ as well as in a human phase I/II clinical study where it was used to deliver the SARS-CoV-2 receptor binding domain (RBD) antigen [clinical trial ID: NCT04839146].

Here, we exploit the cVLP platform to deliver, in parallel, both monomeric and oligomeric HCV sE2 protein. The immunogenicity of these cVLP HCV vaccines was assessed in BALB/c mice, and the vaccine-induced anti-E2 Immunoglobulin G (IgG) antibodies were tested for their capacity to neutralize heterologous HCV strains of clinically relevant genotypes in vitro. cVLP-display only modestly increased induction of anti-E2 IgG and only for monomeric sE2. However, it significantly improved the neutralizing capacity of vaccine-induced IgG, particularly for oligomeric sE2. In fact, while anti-E2 IgG levels were similar for cVLP-coupled monomeric and oligomeric sE2, the latter induced significantly higher levels of bNAbs capable of neutralizing a broad panel of cell-culture infectious HCV (HCVcc). Antigen characterization of the sE2-cVLP vaccine antigens indicated that the AR2A epitope was less exposed/accessible on cVLPs displaying oligomeric sE2 compared to cVLPs displaying monomeric sE2. In addition, competition ELISA suggested that mice immunized with cVLP-coupled oligomeric sE2 induced fewer antibodies specific for AR2A and AR1B, compared with animals immunized with cVLPs displaying monomeric sE2. Thus, this study shows that the modular cVLP vaccine platform enables display of oligomeric forms of HCV sE2, which appear to be superior to induce bNAbs, thus providing important new insights into HCV vaccine design.

## Results

### sE2-cVLP HCV vaccine generation and characterization

**Fig. 1:**
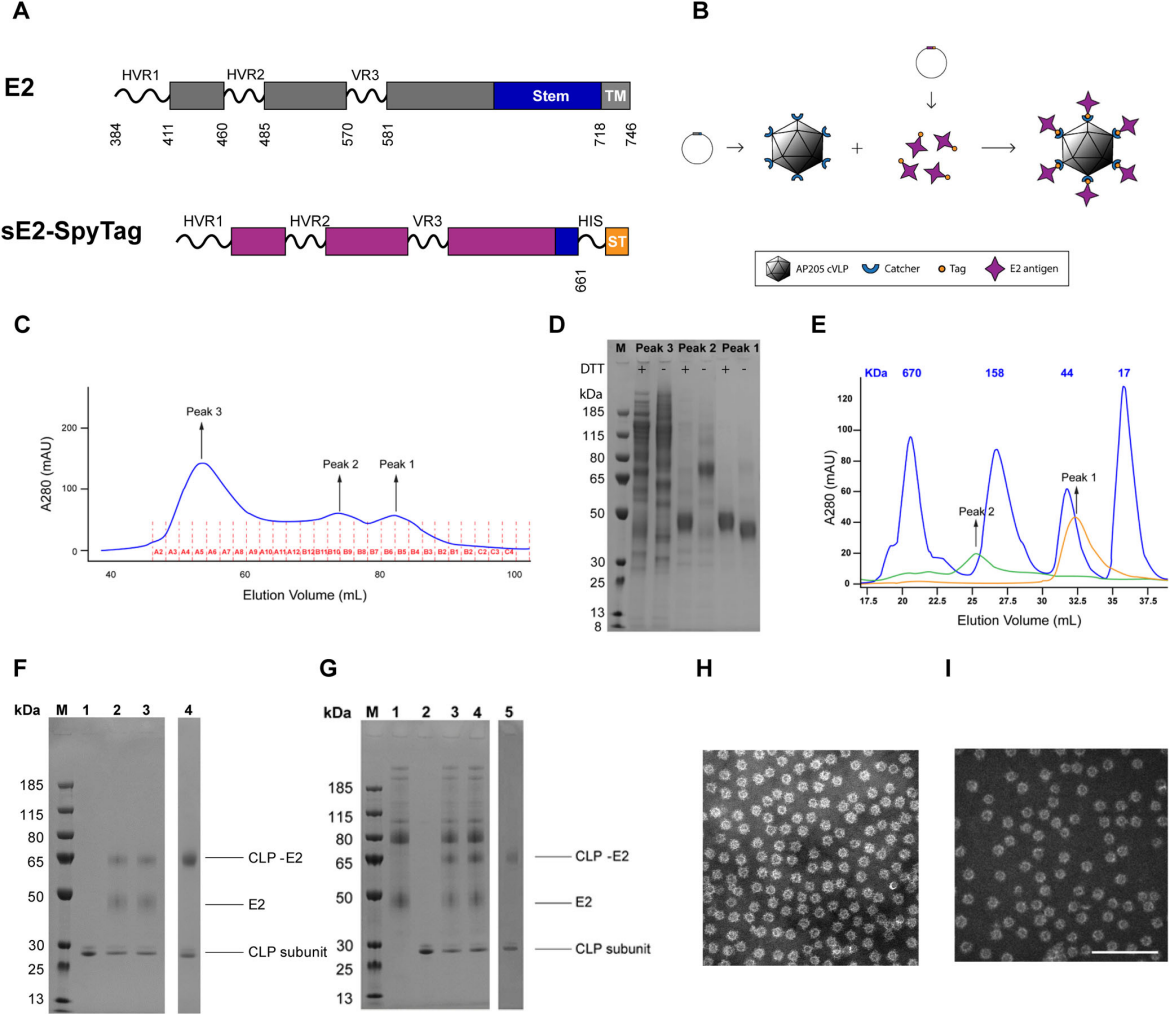
Development of cVLPs populated with monomeric or oligomeric HCV sE2. **A** A schematic illustrating the soluble Con1 (genotype 1b) E2 (sE2) construct used in this study with deletion of part of the stem and the entire transmembrane region (TM) and a C-terminal tag consisting of a 6x polyhistidine (HIS) tag followed by a SpyTag (ST). HVR1 and HVR2, hypervariable region 1 and 2. VR3, variable region 3. **B** A schematic illustrating the principle of the two-component tag/catcher cVLP used to make HCV sE2-cVLPs. **C** Following IMAC purification of sE2 (see Materials and Methods) the protein was further purified using SEC, where it eluted in three distinct peaks. **D** These peaks were evaluated in SDS-PAGE by Coomassie staining either with or without reducing agent (DTT). **E** Peaks 1, 2, and 3 were further evaluated using analytical SEC against proteins of known size. **F** SDS-PAGE gel showing the AP205 CLP prior to monomeric sE2 antigen coupling (Lane 1). Lanes 2 and 3 show the sE2-cVLP vaccine mixture (after 36 h incubation) before (Lane 2) and after (Lane 3) centrifugation (16.000 × *g* for 2 min). Lane 4 shows the final sE2-CLP after removal of excess sE2 antigen by ultracentrifugation. **G** SDS-PAGE gel showing oligomeric sE2 antigen input (Lane 1), the uncoupled AP205 cVLP (Lane 2). Lanes 3 and 4 show the sE2-cVLP vaccine mixture (after 36 h incubation) before (Lane 3) and after (Lane 4) centrifugation (16.000 × *g* for 2 min). Lane 5 shows the final sE2-CLP product after removal of excess sE2 antigen by ultracentrifugation. Densitometric analysis estimates both coupling efficiencies at ~60% (ImageQuantTL). **H** TEM image of monomeric sE2 coupled to cVLPs. **I** TEM image of oligomeric sE2 coupled to cVLPs. Images were taken using similar EM settings and the scalebar represents 200 nm. All the lanes within each gel included in Fig. 1 was processed at the same time.

To develop E2-based cVLP vaccines against HCV, the sE2 antigen (Con1 isolate; genotype 1b) was genetically fused at the C-terminus to SpyTag (Fig. 1A) and was subsequently expressed in *Drosophila* Schneider 2 (S2) insect cells. The SpyTag can spontaneously form a covalent bond with the SpyCatcher protein on the surface of pre-assembled cVLPs (Fig. 1B). Mono- and oligomeric states of the recombinant HCV sE2 were obtained following purification by ion metal affinity chromatography (IMAC) and size separation using size exclusion chromatography (SEC). From the SEC purification it was evident that the IMAC purified protein segregated into three peak fractions (Fig. 1C). Subsequent SDS-PAGE analysis of the SEC fractions indicated that peak 3 contained impurities and high-order oligomeric forms of sE2, whereas the majority of peak 1 and 2 contained the sE2 protein at different oligomeric states (Fig. 1C). Further analytical SEC of purified sE2 against marker proteins of known size, supported that peak 1 represented sE2 monomer (Fig. 1E), whereas the disulfide-mediated sE2 dimers, observed by non-reduced SDS-PAGE (Fig. 1D, peak 2; without DTT), might represent tetramers (referred to as oligomers from this point) in solution (Fig. 1E). Based on these results, both monomeric and oligomeric sE2 were further tested as vaccine antigens.

Monomeric and oligomeric sE2 (with C-terminal SpyTags) were mixed with AP205 cVLPs (each displaying 180 SpyCatcher binding partners), which allowed the HCV sE2 antigens to be directionally attached at high density to the surface of cVLPs (Fig. 1B; SDS-PAGE of the reactions shown for monomeric sE2 in Fig. 1F). Centrifugation of the resulting sE2-cVLP complexes (at 16.000 RCF for two minutes) did not cause precipitation for either monomeric or oligomeric sE2, demonstrating that the vaccines were soluble and stable (Fig. 1F, comparing intensity of CLP-E2 band in lane 3 to lane 2; Fig. 1G, comparing intensity of CLP-E2 band in lanes 4 to 3). Transmission electron microscopy (TEM) imaging showed the presence of monodisperse cVLPs of ~40 nm in diameter for monomeric sE2 (Fig. 1H) and oligomeric sE2 (Fig. 1I), confirming that the cVLPs had formed successfully. These results were supported by dynamic light scattering (DLS) measurement, showing a single population of cVLPs with ~50 nm in diameter and percent polydispersity (%Pd) of 22.6 and 14.6% for monomeric and oligomeric sE2-cVLP vaccines, respectively.

### Coupling HCV sE2 to cVLPs increases the induction of NAbs in immunized mice

**Fig. 2:**
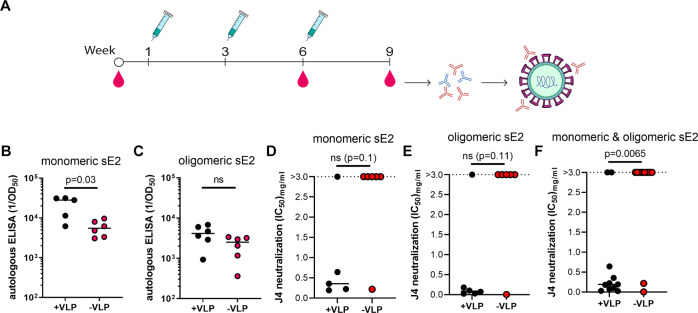
AP205 cVLPs boosts the induction of HCV specific IgG in immunized mice. **A** A schematic illustrating the immunization schedule and serum collection from BALB/c mice. Dose-response ELISAs against either monomeric (**B**) or oligomeric (**C**) sE2 of week 9 mouse serum samples collected from animals immunized either with (**B**) monomeric or (**C**) oligomeric sE2 alone or on cVLPs. ELISAs were done in single replicates using anti-mouse IgG coupled to HRP and subsequently a colorimetric TMB substrate signal was detected. Binding results were assessed by four parameter curve fitting to calculate OD_50_ values. Data is represented with median value of inverse OD_50_ values of purified IgG from individual animals. **D**, **E** Dose-response neutralization of HCV recombinant J4 (genotype 1b) HCVcc was done in three replicates using mouse IgG and was evaluated in FFU reduction assays on Huh7.5 cells. Following 48 h of infection the cells were fixed and FFUs were visualized using HCV anti-NS5A antibody followed by anti-mouse antibody coupled to HRP followed by DAB staining of FFUs. Results were normalized to non-infected control cells and neutralization was assessed by four parameter curve fitting to calculate IC_50_ values. If 50% neutralization was not reached at the highest tested dose then IC_50_ values were interpolated if neutralization at highest dose reached a minimum of 30% and increased to at least 50% at 3 mg/ml. Data is represented with median value of inverse IC_50_ values of purified IgG from individual animals. **F** The IC_50_ values from panels **D** and **E** were pooled into groups where antigen was either coupled to VLPs or not. Statistical significance testing was done using non-parametric Mann-Whitney tests (Graphpad Prism 9.2.0) and the significance threshold was set at 95%.

Mice were immunized using a prime-boost-boost regimen with either monomeric or oligomeric HCV sE2 antigens, alone or displayed on the surface of the cVLPs, as described above. Animals were euthanized at three weeks after the final booster immunization (week 9) and IgG was purified and used for in vitro testing of binding and neutralization (Fig. 2A). To assess the immunogenicity of vaccines containing monomeric and oligomeric sE2, respectively, sE2 specific antibody titers were measured by ELISA. Specifically, monomeric sE2 was used for capture of anti-E2 IgG induced by cVLPs displaying monomeric sE2. Similarly, oligomeric sE2 was used for capture of anti-E2 IgG induced by cVLPs displaying oligomeric sE2. cVLPs displaying monomeric sE2 antigen produced significantly higher antibody titers (*p* = 0.03, geometric mean of reciprocal of 50% max optical density titer measured at 17.998) compared to the non-displayed sE2 antigen (geometric mean of titer 5.326, Fig. 2B) used for benchmarking. For oligomeric sE2 the trend was similar, but the antibody titers induced by the oligomeric sE2-cVLP vaccine (geometric mean of titer 3.500) were not statistically significantly different to those induced by the non-displayed, oligomeric sE2 antigen (geometric mean of titer 1.700, Fig. 2C). To directly compare the level of sE2-specific antibodies induced by cVLPs displaying monomeric and oligomeric sE2, respectively, serum samples taken three weeks after the 1st booster immunization (week 6) were analyzed in the same ELISA, using either monomeric or oligomeric sE2 as capture antigen. Here, we observed no difference in the ELISA titers (Supplementary Fig. 1A, B).

To measure the vaccine-relevant functionality of the induced IgG following immunization with monomeric/oligomeric sE2-cVLP HCV vaccines or non-displayed controls, HCV neutralization assays were conducted using the genotype-matched HCVcc, J4 (genotype 1b). Virus stocks were generated, and the envelope protein sequences confirmed as previously described^38^. Focus forming unit (FFU) reduction assays were performed by pre-incubating a dilution series of total IgG purified from immunized mouse serum with J4 HCVcc recombinant virus, prior to infection of HCV-permissive Huh7.5 cells. Specifically, when the sE2 antigens (monomeric and oligomeric) were displayed on cVLPs we observed neutralization in 5 out of 6 animals, as compared with 1 out of 6 animals for the non-cVLP-displayed sE2 antigens. While statistical significance for the benefit of displaying sE2 on cVLPs was not reached for monomeric (*p* = 0.1) or for oligomeric sE2 (*p* = 0.11) when analyzed alone (Fig. 2D, E), the benefit was highly significant when the cVLP-coupled samples were pooled and compared to non-displayed samples (*p* = 0.0065; Fig. 2F). Intriguingly we also observed a trend for increased induction of NAbs using cVLP-coupled oligomeric sE2 compared with cVLP-coupled monomeric sE2.

### Oligomeric HCV sE2 displayed on cVLPs induces cross-genotype-reactive bNAbs

**Fig. 3:**
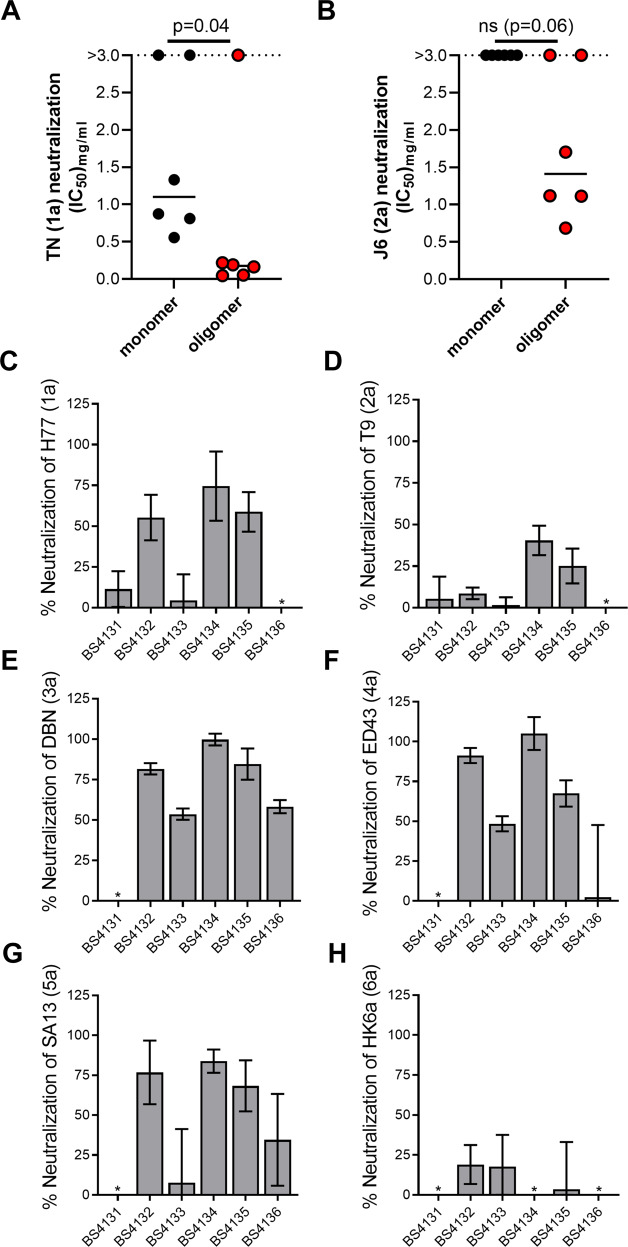
Oligomeric HCV sE2 coupled to cVLPs elicits cross-reactive neutralizing antibodies against cross-genotypic HCVcc panel. Mouse IgG from BALB/c mice immunized with cVLPs coupled either to monomeric or oligomeric sE2 was used in three replicates in dose-response neutralization of HCV recombinants (**A**) TN (genotype 1a) or (**B**) J6 (genotype 2a) HCVcc in FFU reduction assays on Huh7.5 cells. Following 48 h of infection the cells were fixed and FFUs were visualized using HCV anti-NS5A antibody following by anti-mouse antibody coupled to HRP followed by DAB staining of FFUs. Results were normalized to non-infected control cells and neutralization was assessed by four parameter curve fitting to calculate IC_50_ values. If 50% neutralization was not reached at the highest tested dose then IC_50_ values were interpolated if neutralization at highest dose reached a minimum of 30% and increased to at least 50% at 3 mg/ml. Data is represented with median value of inverse IC_50_ values of purified IgG from individual animals. Statistical significance testing was done using non-parametric Mann–Whitney tests (Graphpad Prism 9.2.0) and the significance threshold was set at 95%. Mouse IgG from BALB/c mice immunized with oligomeric HCV sE2 was used at a single dose of 0.4 mg/ml in three replicates to neutralize Huh7.5 cell infection of HCVcc recombinants **C** H77 (genotype 1a), **D** T9 (genotype 2a), **E** DBN (genotype 3a), **F** ED43 (genotype 4a), **G** SA13 (genotype 5a), or **H** HK6a (genotype 6a). Results were normalized to non-infected control cells and is shown as percent neutralization with standard deviation. BS4131-BS4136, refers to individual BALB/c mouse serum samples taken at week 9 from which the IgG was extracted. *No measurable neutralization.

To further test whether cVLP-displayed oligomeric sE2 induced higher levels of bNAbs than cVLP-displayed monomeric sE2, we performed dose-response FFU reduction assays against HCVcc of isolates TN (genotype 1a) and J6 (genotype 2a). Here, we observed that oligomeric sE2 induced significantly higher TN neutralizing titers (*p* = 0.04), as well as increased neutralization of J6, which was close to being statistically significant (*p* = 0.06) (Fig. 3A, B). J6 was generally much less neutralized than TN, which could be due to either epitope differences between the antigen isolate (Con1; genotype 1b) and J6 (genotype 2a) or be due to the fact that J6 is generally a hard-to-neutralize strain^39,40^. For J6, only cVLP-displayed oligomeric sE2 induced any quantifiable neutralization. Thus, these data indicated that cVLP-coupled oligomeric sE2 was a superior HCV vaccine antigen.

To further test the capacity of oligomeric sE2 coupled to cVLPs to induce bNAbs in mice, we performed additional HCVcc neutralization experiments. However, due to limited sample availability we were unable to perform dose-response neutralization experiments and instead performed single moderate dose IgG neutralization at 0.4 mg/ml against a panel of viruses representing HCV genotypes 1–6; H77 (genotype 1a), T9 (2a), DBN (3a), ED43 (4a), SA13 (5a) and HK6a (6a). IgG from the majority of animals immunized with cVLP-displayed oligomeric sE2 contained bNAbs against H77, DBN, ED43, and SA13 (Fig. 3C, E–G). The low effect against the genotype 2a virus, T9, at 0.4 mg/ml IgG, was not unexpected (Fig. 3D), as we observed a mean IC_50_ value at or greater than 1.8 mg/ml for the genotype 2a virus, J6 (Fig. 3B). Also, although the genotype 6a isolate, HK6a, is very sensitive to NAbs^38^, we did not observe convincing neutralization, indicating that perhaps this antigen does not induce effective NAbs against genotype 6 (Fig. 3H). Overall, we concluded that cVLP-displayed oligomeric sE2 was capable of inducing robust bNAb responses against the majority of tested strains.

### cVLPs displaying monomeric and oligomeric sE2 exhibit different antigenicity and induce anti-E2 IgG responses with different specificity

**Fig. 4:**
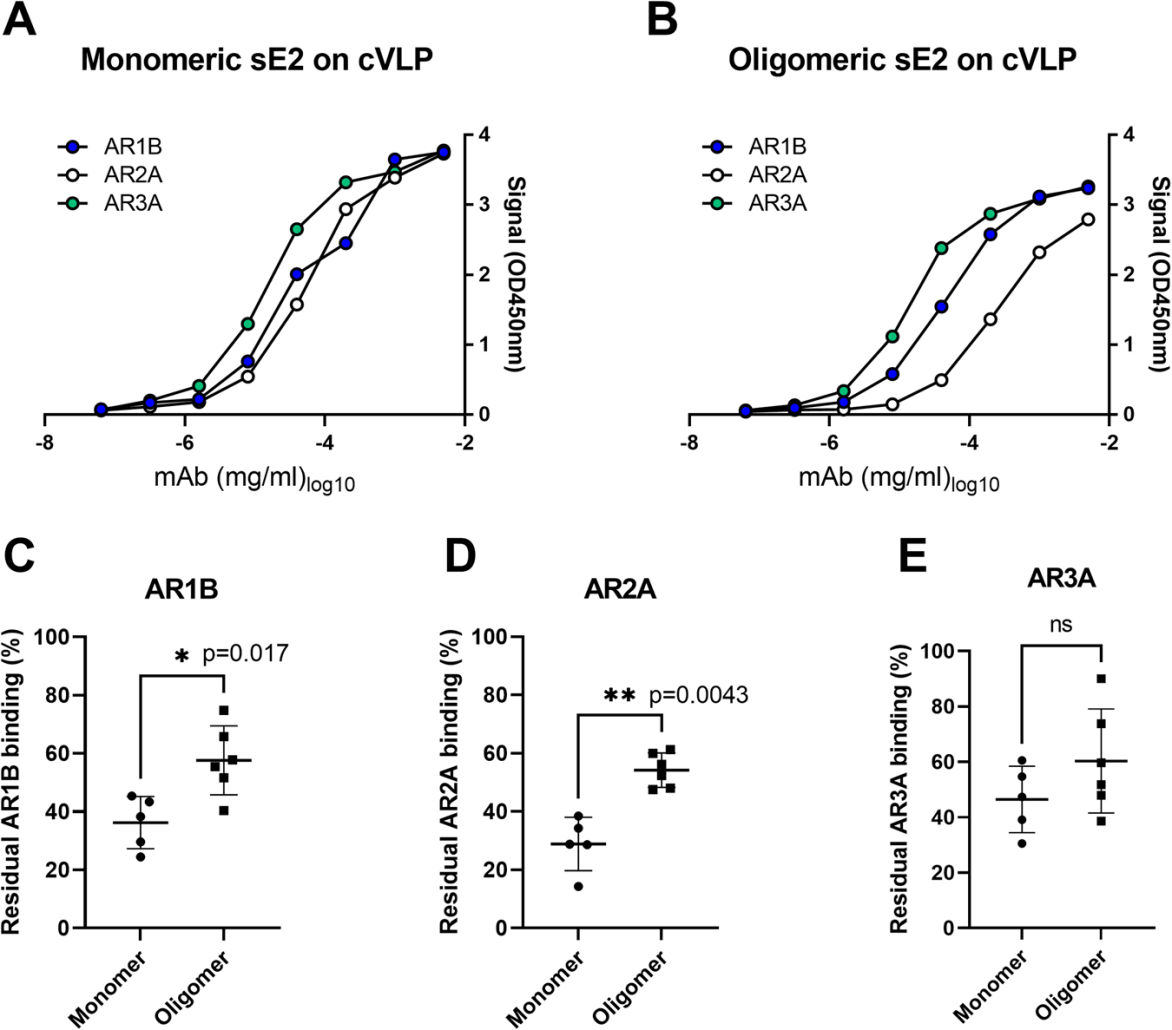
Oligomeric sE2-cVLPs present less AR2A epitopes than monomeric sE2-cVLPs and induces significantly less AR1B- and AR2A-specific antibodies in mice. Dose-response ELISAs against cVLPs coupled either to (**A**) monomeric or (**B**) oligomeric sE2 using human monoclonal anti-HCV antibodies AR1B, AR2A, or AR3A. ELISAs were done in single replicates using anti-human IgG coupled to HRP and subsequently, a colorimetric TMB substrate signal was detected. **C**–**E** Competition ELISAs were performed using week 6 mouse sera in two replicates in a 3-fold dilution series, starting at 1:200, to block binding of AR1B, AR2A, or AR3A (see Supplementary Fig. 2 for full dose-response analysis). Residual binding was calculated by comparing to wells without blocking serum and converted to percent. Values represent mean and the error bars represent the standard deviation. Pre-immune sera were shown to have no inhibitory effect for the three tested antibodies (Supplementary Fig. 2). Statistical significance testing was done using non-parametric Mann–Whitney tests (Graphpad Prism 9.2.0) with a significance threshold set at 95%.

To investigate antigenic differences between monomeric and oligomeric sE2 displayed on cVLPs, titration series of human monoclonal NAbs AR1B, AR2A, and AR3A were tested for binding to immobilized sE2-cVLP complexes (either monomeric or oligomeric sE2) in ELISA. This showed that the monoclonal NAbs, which target the AR1B and the cross-genotype conserved AR3A epitope, respectively, bind similarly to cVLPs displaying monomeric or oligomeric sE2 (Fig. 4A, B). However, the monoclonal NAb, targeting the AR2A epitope (known to be poorly accessible on HCV virus particles), showed decreased binding to cVLP-coupled oligomeric sE2 (Fig. 4A, B).

We next performed competition ELISAs using serum drawn at three weeks after the 1st booster immunization (week 6) with either monomeric or oligomeric sE2 coupled to cVLPs. Here, we observed dose-response inhibition of binding for the three tested antibodies: AR1B, AR2A, and AR3A (Supplementary Fig. 2A–I). In addition, statistical analysis at the highest tested dose revealed similar levels of AR3A antibodies, but significant reduction in the induction of AR2A (*p* = 0.0043) and AR1B (*p* = 0.017) antibodies by oligomeric sE2 coupled to cVLPs (Fig. 4C–E). Taken together, this suggests that oligomeric sE2 coupled to cVLPs has an altered, and likely advantageous, antigenic profile compared with monomeric sE2 coupled to cVLPs.

## Discussion

Combating the continued spread of HCV hinges on developing a vaccine that prevents chronicity. Here, we employ a two-component vaccine platform based on cVLPs to deliver monomeric and oligomeric HCV sE2, respectively. Head-to-head comparison of vaccine formulations (all including Addavax adjuvant) showed that cVLP display had no significant effect on the overall immunogenicity of the sE2 antigen. However, mice that were immunized with cVLP-displayed sE2 induced anti-E2 IgG with an improved capacity for neutralizing HCV in vitro. Furthermore, this effect was greater for cVLPs displaying oligomeric sE2 than for cVLPs displaying monomeric sE2, indicating that cVLP-display of oligomeric E2 assemblies may represent a superior HCV vaccine design. This superiority may be linked to an altered antigen profile of cVLPs displaying sE2 oligomers. In support of this hypothesis, our data indicate that AR2A-specific epitopes are less exposed on cVLPs displaying oligomeric sE2 and that these appear to induce less AR2A- and AR1B-specific antibodies compared to cVLP displaying monomeric sE2.

We show proof-of-concept that displaying sE2 on cVLPs boosts the induction of HCV bNAbs, which is in agreement with the findings of others using single-component VLP/NP approaches for HCV (i.e., where the antigen is genetically fused to the particle-forming protein)^30,33^. Importantly, the two-component (i.e., modular) nature of the employed cVLP vaccine design permits the comparison of monomeric and multimeric antigens. Modifications of HCV sE2 can increase the ability of the protein to induce bNAbs^26–31^ and studies have shown that some E2 antigen designs (E2 receptor-binding domain lacking three variable regions) can form disulfide-linked high-molecular-weight (HMW) E2 proteins that, when used as vaccine antigens, elicit antibodies with an increased neutralizing activity compared with corresponding monomeric species^26,29,41^. Interestingly, the increased ability of these HMW E2 antigens for generating bNAbs was shown to be correlated with occlusion of the non-neutralizing face of E2 in the oligomeric assembly state. Here, we identify an SEC fraction containing oligomeric sE2 which, in non-reducing SDS-PAGE, migrates as a sE2 dimer and that according to analytical SEC has the size of a sE2 tetramer. This enabled a direct comparison of the antigenic properties of oligomeric versus monomeric sE2 delivered by the same cVLP vaccine platform. Interestingly, we found that oligomeric sE2 induced cross-genotype reactive bNAbs capable of neutralizing all epidemiologically relevant genotypes, with the possible exception of genotype 6a, although an effect might have been observed at higher IgG concentrations. In comparison, cVLPs displaying monomeric sE2 induced inferior NAb responses. Antigenic characterization revealed that the AR2A epitope, which is known to be neutralizing, but poorly accessible on infectious virus particles^24,25^, was less antigenic on oligomeric sE2 displayed on cVLPs, compared with monomeric sE2 on cVLPs. This difference may be linked to the results obtained by competition ELISAs, showing that animals immunized with oligomeric sE2 coupled to cVLPs have less antibodies competing with the human monoclonal anti-AR2A and AR1B antibodies. Collectively, these data underline antigenic differences between monomeric and oligomeric sE2, which may be directly involved in the improved induction of bNAbs by oligomeric sE2 on cVLPs.

Thus, our data suggests that cVLP display of oligomeric sE2 represents a promising design for an HCV vaccine. Future studies should clarify whether additional E2 antigens, based on sequences from other HCV isolates, will exhibit similar qualitative differences when delivered in monomeric and oligomeric forms, respectively. Also, optimization of the gene design and subsequent protein purification procedures could lead to further antigen improvements and is needed to enable large-scale antigen production. Furthermore, future HCV vaccine studies could benefit from recently developed HCV pseudo-particle or HCVcc virus panels, representing much of the diversity in HCV neutralization sensitivity^40,42^, which will provide a platform for comparing the vaccine potential across different approaches developed in different labs.

Thus, we show proof-of-concept that the two-component cVLP-based vaccine platform boosts the induction of HCV bNAbs. Importantly, the two-component approach permitted testing oligomeric sE2-cVLPs, which showed the induction of statistically significant higher levels of bNAbs, compared with monomeric sE2-cVLPs. This is of particular interest as we and others have shown how sE2 or detergent-extracted E1E2 protein only partly recapitulates the behavior of virion-associated E1/E2 complexes^43–47^. Thus, our findings suggest that future complex HCV envelope antigens, such as iterations of the recently described native-like sE1E2 antigens^15^ or the sE1E2 permuted antigens^48^, could benefit from being combined with the cVLP approach described here, or similar two-component VLP platforms, to boost bNAb responses in animals and humans.

In summary, by decorating cVLPs with oligomeric HCV sE2 protein we are able to greatly boost the induction of bNAbs. These data offer important insights to be used for the design of a protective HCV vaccine.

## Methods

### Recombinant antigen expression

Production of soluble, recombinant E2 (sE2) antigen was based on the Con1 (genotype 1b) isolate sequence (amino acids 384–661)^35,49^, which was truncated in the C-terminal transmembrane stem region. The gene sequence was modified to contain an N-terminal BIIP sequence as well as a C-terminal 6x poly-histidine (HIS) tag followed by a SpyTag (Fig. 1A). The final gene had flanking restriction sites (EcoRI and NotI) added to the N- and C-terminus and was codon optimized for expression in *Drosophila* Schneider 2 (S2) insect cells. The gene was synthesized by GeneArt (Thermo Scientific) and subcloned into the pExpreS^2^-1 (zeocin resistance) vector (ExpreS^2^ion Biotechnologies, Horsholm, Denmark) using EcoRI and NotI restriction sites.

The S2 cell culture supernatant was concentrated five times and buffer exchanged into 20 mM Tris, 0.5 M NaCl, pH 7.9 using a tangential flow filtration system (Quixstand Benchtop system, MWCO 10 kDa). Concentrated and buffer exchanged supernatant was loaded onto 5 mL HisTrap^TM^ HP columns for ion metal affinity chromatography (IMAC) and purified using the above buffer with 60 mM Imidazole and increasing to 0.5 M Imidazole in the elution buffer for step elution of the bound protein. The elution peak was further purified by size exclusion chromatography (SEC) on a 200 pg Sephadex column. Post purification SEC fractions of peak 1 and 2 (Fig. 1C) were used for subsequent immunization studies as monomeric and oligomeric sE2 antigens, respectively.

### AP205 cVLP production

The SpyCatcher-AP205 cVLP is assembled from fusion proteins comprising the major capsid protein of the Acinetobactor phage AP205 (Gene ID: 956335) and SpyCatcher (Genbank: AFD50637, aa 24-139), respectively. Specifically, the SpyCatcher-AP205 sequence was constructed by fusing the SpyCatcher sequence to N-terminus of the AP205 coat protein, using a flexible linker (Gly-Gly-Ser-Gly-Ser), and subsequently adding flanking NcoI and NotI restriction sites at the N- and C-termini, respectively. The gene sequence was finally codon-optimized for recombinant expression in *E. coli* and synthesized by (GeneArt® Life Technologies, Germany). The synthetic gene was cloned into a pET-15b vector and transformed into competent One Shot® BL21 Star™ (DE3) cells (Thermo Scientific). Recombinant protein expression was done in 3 L shake flasks containing 400 mL 2xYT media (100 µg/mL ampicillin). The bacterial culture was incubated at 37 °C for three hours (OD600 = 0.6) at which point cells were induced with 1 mM IPTG. The induced culture was then incubated for additional 16 h at 20 °C. Bacterial cells were harvested by centrifugation (10,000 × *g*) and the pellet was resuspended in 1× PBS (pH = 7.2) and lysed by sonication at 80% power with 5 pulsations for 2 × 5 min on ice. The cleared bacterial lysate was finally purified by ultracentrifugation (UC) using an Optiprep™ (Sigma) step (23, 29, and 35%) gradient. Specifically, 1.2 ml lysate was loaded on top of the density step-gradient and spun at 307.900 RCF (SW60Ti rotor, Beckmann Coulter) for 3.30 h at 16 °C. The UC sample was then divided into smaller fractions (~200 µl), which were analyzed for protein content by SDS-PAGE. Fractions containing SpyCatcher AP205 cVLPs were finally pooled and dialyzed against 20 mM Tris, 0.5 M NaCl buffer using a 1000 kDa MWCO SpectraPor Membrane.

### Vaccine production

Purified, SpyTagged monomeric or oligomeric sE2 protein was mixed with SpyCatcher-AP205 cVLP at molar ratio 2:1 (antigen per cVLP binding site) and incubated for 36 h at 4 °C to generate sE2-cVLPs (Fig. 1B). Analysis of the vaccine mixture was done by SDS-PAGE. Briefly, 10 µL of the vaccine sample was mixed with 2 µL SDS loading dye (with or without Dithiothreitol). After heating the sample at 95 °C for 5 min, it was added (with a molecular size marker) to a NuPAGE Bis-Tris gel and run for 1 h at 170 V. The SDS-PAGE gel was finally stained with coomassie blue. SDS-PAGE densitometric analysis (ImageQuantTL) was used to determine the coupling efficiency of the SpyTag-SpyCatcher interaction by dividing the intensity of the cVLP band in the coupling lane with the intensity of the cVLP band in an input lane containing the equivalent amount of cVLP used in the coupling reaction and multiplying this value with 100. Excess unbound antigen was removed by repeating the ultracentrifugation step. sE2-cVLP containing fractions were pooled and dialyzed against 20 mM Tris, 0.5 M NaCl, pH7.9 as above and bacterial endotoxins were removed from the non-displayed and cVLP-displayed vaccines by a phase separation method using Triton X-114 detergent. Specifically, Triton X-114 was added in a 1:100 volume ratio to the protein sample, which had been chilled on ice. The sample was then mixed by vortexing and incubated on ice for 5 min. Subsequently, the sample was incubated at 37 °C for 5 min and centrifuged for 1 min at 16.000 × *g* at 37 °C. After separating the supernatant from the pellet, the above procedure was repeated using only the supernatant. Triton X-144 was finally removed by dialysis. All gel electrophoresis lanes shown in Coomassie stains of SDS-PAGE were run at the same time.

### Transmission electron microscopy (TEM)

For TEM imaging, monomeric and oligomeric sE2-cVLP vaccines samples were diluted to 0.2 mg/mL in PBS and adsorbed to 200-mesh-carbon-coated grids, which were stained with 2% phosphotungstic acid (pH = 7.0) for 1 min. The negatively stained sample was finally analyzed in a CM100 BioTWIN electron microscope (Phillips) at an accelerating voltage of 80 kV. Pictures were taken using an Olympus Veleta camera.

### Dynamic Light Scattering (DLS)

DLS was used to investigate the distribution of cVLP particle sizes. Samples were spun for 2.5 min at 16.000 RCF before analysis. Twenty repeated measurements were acquired for each sample (at 658 nm, 25 °C, WYATT Technology, DynaPro NanoStar). The diameter was calculated from the hydrodynamic radius of the particles along with percent polydispersity (%Pd).

### Animal immunizations

Mice were immunized with monomeric or oligomeric HCV sE2, alone or displayed on AP205 cVLP, to evaluate the immunogenicity and capacity for inducing NAbs of the different vaccines. Each immunization group contained six female BALB/c mice (8 weeks old) that were immunized intramuscularly in the thigh at weeks 1, 3, and 6. Mouse blood samples were taken at weeks 0 (pre-bleed), 6, and 9 (Fig. 2A). Per immunization, each mouse received 1.5 µg of either non-displayed or cVLP-displayed sE2 antigen formulated 1:1 (vol/vol) in Addavax (Invivogen).

### HCV sE2 specific serum IgG levels

To determine the level of HCV sE2-specific antibody titers raised following immunization with both monomeric and oligomeric variants of the sE2 antigen displayed on cVLP or non-displayed, an enzyme-linked immunosorbent assay (ELISA) was used. Specifically, 96-well microtiter plates (Nunc MaxiSorp) were coated over night at 4 °C with monomeric or oligomeric sE2 with C-terminal HIS-Tag (0.1 µg per well) produced in insect cells. PBS buffer +0.5% skim milk powder (w/v) was added for blocking of the plates. PBS was used for all washing steps. Secondary goat anti-mouse IgG Horseradish peroxidase (Novex) (1:2000 dilution) was used in as secondary antibody and 3,3′,5,5′-Tetramethylbenzidine (TMB) as substrate for developing the plates. The colorimetric reaction was terminated with 0.2 M H_2_SO_4_ and the signal was measured at OD 450 nm. IgG titers were normalized across plates using a positive control and antibody titers were calculated by determining OD_50_ (after modeling the serum dilution curves in Graphpad Prism (9.2.0) software using four parameter non-linear regression curve fitting).

### Competition ELISA against AR1B, AR2A and AR3A

96-well microtiter plates (Nunc MaxiSorp) were coated with monomeric sE2 at 0.4 µg/ml ON at 4 °C. The following day, the plates were washed with PBS 0.1%Tween20 and blocked using PBS buffer with 1% skim milk powder (w/v) and 5% BSA (BSK). Three-fold dilution series of serum were made in PBS 0.1%Tween20 starting at 1:200 and incubated with the plates ON at 4 °C. The following day, human monoclonal antibodies AR1B, AR2A, and AR3A were used at 50% binding concentrations and incubated in BSK for 30 min at room temperature. After washing in PBS 0.1%Tween20 sheep anti-human secondary antibody coupled to HRP (NA933V) was incubated at 1:1000 dilution in BSK for 1 h at room temperature. 3,3′,5,5′-Tetramethylbenzidine (TMB) was used as substrate for developing the plates and the colorimetric reaction was terminated with 2 M H_3_PO_4_ and the signal was measured at OD 450 nm.

### Antigenic sE2-cVLP characterization by ELISA

96-well microtiter plates (Nunc MaxiSorp) were coated with cVLPs displaying monomeric or oligomeric sE2 (0.1 µg per well). Dilution series of HCV E2-specific human monoclonal antibodies AR1B, AR2A, or AR3A^24^ were subsequently tested for binding to the immobilized E2-cVLP complexes to probe epitope accessibility. PBS buffer +0.5% skim milk powder (w/v) was used for blocking the plates and for serum dilutions. PBS was used for all washing steps. Secondary goat anti-human IgG Horseradish peroxidase (Agilent Technologies) was used as secondary antibody (1:5000 dilution) and TMB as substrate for developing the plates. The colorimetric reaction was terminated with 0.2 M H_2_SO_4_ and the signal was measured at OD 450 nm.

### Mouse IgG purification and quantification

For the neutralization experiments, mouse IgG was purified from serum taken at 3 weeks after final booster immunization (week 9). Polyprep® chromatography columns (Bio-Rad) were flushed with 20 mM phosphate buffer, pH 7.2, and packed with Gammabind Plus Sepharose (GE Healthcare). One column volume of 20 mM phosphate buffer, pH 7.2 was used to rinse the column material before loading the individual, 0.2 µm filtered mouse serum samples through the column material five times. Bound IgG was washed by rinsing the column with 100 mM Citric Acid (pH 4). After the washing procedure, mouse IgG was released by eluting with 100 mM Citric Acid (pH 2.7) in a neutralization buffer (to a final pH of 7.5 using 1 M Tris-HCl, pH 9). Eluted IgG samples were dialyzed (MWCO 12–14 kDa, Spectrum Labs) into PBS and concentrated with Vivaspin 6, MWCO 50 kDa. Finally, concentrations were measured by the IgG setting on the Nanodrop2000 (Thermo Scientific).

### In vitro HCV neutralization assay

7 × 10^3^ of HCV permissive Huh7.5 cells were plated per well in poly-D-lysine 96-well plates and incubated for 24 h. All neutralization assays were done with purified IgG instead of full serum to avoid non-IgG effects from mouse serum components. The following day, a dilution series of mouse-derived antibodies (at the highest dose of 0.5–1 mg/ml) was made using DMEM (Gibco) supplemented with 10% fetal calf serum (Sigma) and penicillin/streptomycin (Sigma) (full medium). This was incubated in a total volume of 10 μl with cell-culture infectious JFH1-based Core-NS2 recombinant HCVcc stocks, referred to by the isolate name of the envelope proteins: H77 (genotype 1a), TN (genotype 1a), J4 (genotype 1b), J6 (genotype 2a), T9 (genotype 2a), DBN (genotype 3a), ED43 (genotype 4a), SA13 (genotype 5a) and HK6a (genotype 6a)^38,50–53^, corresponding to a final read-out of 50–200 FFU per well. The virus/antibody mixes were, along with eight replicates of virus only, incubated for 1.5 h at 37 °C before addition of an additional 20 μl of full medium and incubation with Huh7.5 cells for 2.5 h at 37 °C in 5% CO_2_. Cells were washed, and fresh medium was added prior to incubation for a total infection time of 48 h before fixation. Following fixation, the cells were incubated with anti-mouse Fab fragments (Jackson Immuno Research) to block the Fc region of the mouse antibodies.

Cells were stained by overnight incubation with NS5A-specific antibody 9E10 in PBS buffer with BSK. Cells were washed three times in PBS buffer with 0.1%Tween20 and incubated 1 h at room temperature with secondary antibody Anti-mouse IgG, Horseradish Peroxidase (Amersham Biosciences) at a dilution of 1:2000 and visualized by DAB staining (VWR). FFUs were counted on an ImmunoSpot series 5 UV analyzer (CTL Europe GmbH) with customized software. The mean FFU count of 8 negative-control wells was subtracted from FFU counts in experimental wells. The data were normalized to 8 replicates of virus only and analyzed using three or four parameters curve fitting in GraphPad Prism 9.2.0, bottom set to 0, top set to 100.

### Statistical analysis

All statistical analysis and graphs were prepared using the GraphPad Prism 9.2.0 software. The non-parametric, two tailed, Mann–Whitney Rank Sum Test unpaired comparison between immunization groups was used to test for statistical difference amongst different vaccination groups; statistical significance was defined as *p* < 0.05.

### Reporting summary

Further information on research design is available in the Nature Research Reporting Summary linked to this article.

## Supplementary information

Supplementary data file

REPORTING SUMMARY

## Data Availability

All data is freely available upon request. Please contact Jannick Prentoe (Jprentoe@sund.ku.dk).
