# Supplementary material for: Two-component vaccine consisting of virus-like particles displaying hepatitis C virus envelope protein 2 oligomers
Source: NPJ Vaccines. 2022 Nov 15;7:148. doi: 10.1038/s41541-022-00570-1 (PMC9665036; doi:10.1038/s41541-022-00570-1)
Supplement: Supplementary file 1 — Supplementary data file [file 41541_2022_570_MOESM1_ESM.pdf]

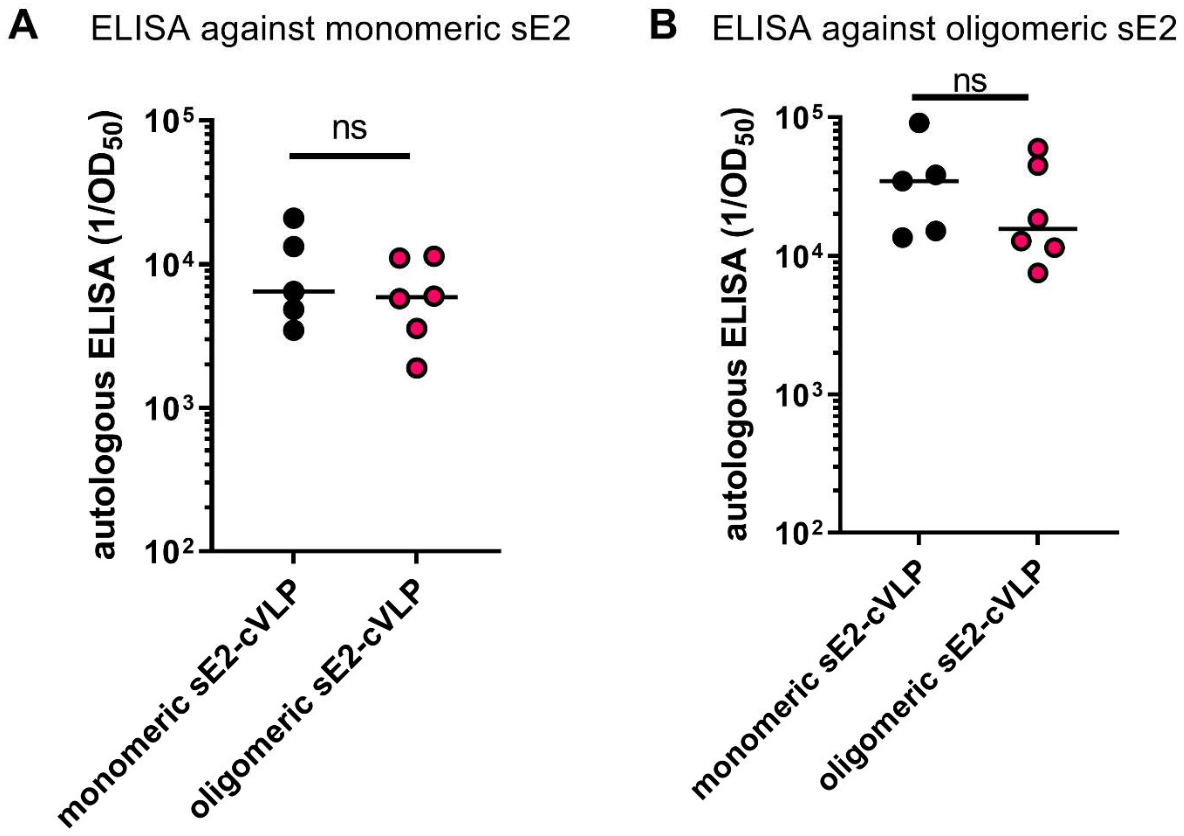

Supplementary Figure 1. Week 6 serum from mice immunized with cVLP-displayed monomeric and oligomeric E2 show similar anti-E2 ELISA titers irrespective of using monomeric or oligomeric E2 as capturing antigen. A-B) ELISAs using either monomeric (A) or oligomeric (B) sE2 as capture antigen were performed on serum samples obtained three weeks after prime-boost vaccination (week 6) with cVLPs displaying monomeric or oligomeric sE2, respectively. ELISAs were done in single replicates using secondary HRP-conjugated anti-mouse IgG and colorimetric TMB substrate for detection. Binding results were assessed by four parameter curve fitting to calculate OD_{50} values. Data is represented with median value of inverse OD_{50} values of purified IgG from individual animals.

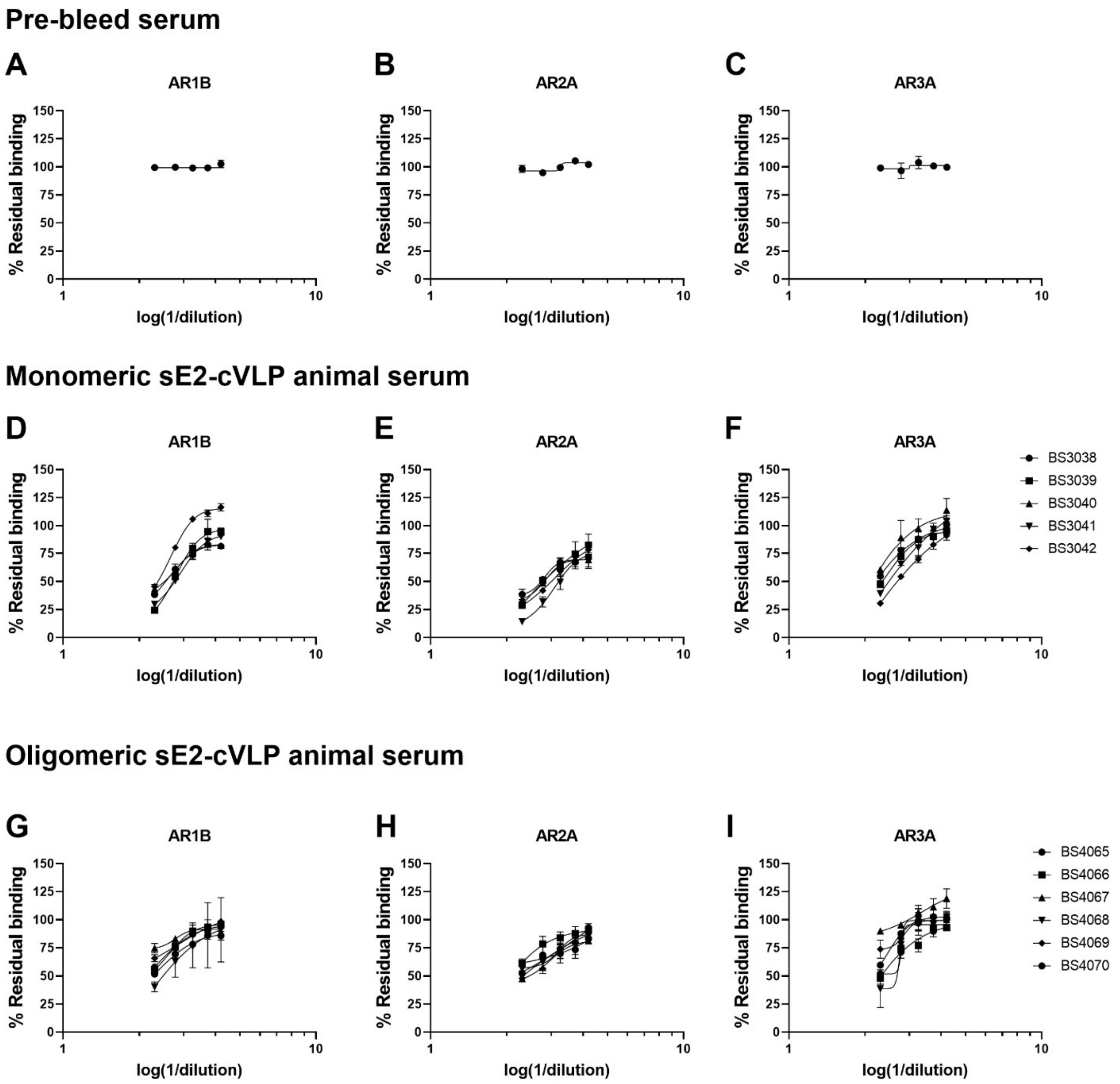

Supplementary Figure 2. Week 6 serum samples from mice immunized with cVLP-displayed monomeric and oligomeric E2 competed with human monoclonal antibodies AR1B, AR2A and AR3A. Maxisorp 96-well plates were coated at 0.4 µg/ml ON at 4°C. Then the wells were incubated with three-fold dilution series starting at 1:200 dilutions of pre-immune serum (A-C) or serum taken at week 6 from animals vaccinated with either monomeric (D-F) or oligomeric (G-I) sE2 coupled to cVLPs. Finally, replicate wells preincubated with serum or with no preincubation were incubated with human monoclonal antibody AR1B, AR2A or AR3A. Human antibody binding was measured

by incubation with sheep anti-human-IgG coupled to HRP and colorimetric TMB substrate for detection. Data was normalized to 8 replicates of wells incubated with human monoclonal antibody without pre-incubation with serum. Residual binding was calculated by comparing to wells without blocking serum and converted to percent. Error bars represent standard deviation. BS3038-3042 and BS4065-4070, refers to individual BALB/c mouse serum samples taken at week 6.
