# Supplementary material for: Two-component vaccine consisting of virus-like particles displaying hepatitis C virus envelope protein 2 oligomers
Source: NPJ Vaccines. 2022 Nov 15;7:148. doi: 10.1038/s41541-022-00570-1 (PMC9665036; doi:10.1038/s41541-022-00570-1)
Supplement: Supplementary file 2 — REPORTING SUMMARY [file 41541_2022_570_MOESM2_ESM.pdf]

nature portfolio

Corresponding author(s): Jannick Prentø

Last updated by author(s): 04-03-2022

Reporting Summary

Nature Portfolio wishes to improve the reproducibility of the work that we publish. This form provides structure for consistency and transparency in reporting. For further information on Nature Portfolio policies, see our Editorial Policies and the Editorial Policy Checklist.

Statistics

For all statistical analyses, confirm that the following items are present in the figure legend, table legend, main text, or Methods section.

n/a	Confirmed	
☐	☒	The exact sample size (n) for each experimental group/condition, given as a discrete number and unit of measurement
☐	☒	A statement on whether measurements were taken from distinct samples or whether the same sample was measured repeatedly
☐	☒	The statistical test(s) used AND whether they are one- or two-sided<br>*Only common tests should be described solely by name; describe more complex techniques in the Methods section.*
☒	☐	A description of all covariates tested
☐	☒	A description of any assumptions or corrections, such as tests of normality and adjustment for multiple comparisons
☐	☒	A full description of the statistical parameters including central tendency (e.g. means) or other basic estimates (e.g. regression coefficient) AND variation (e.g. standard deviation) or associated estimates of uncertainty (e.g. confidence intervals)
☐	☒	For null hypothesis testing, the test statistic (e.g. F, t, r) with confidence intervals, effect sizes, degrees of freedom and P value noted<br>*Give P values as exact values whenever suitable.*
☒	☐	For Bayesian analysis, information on the choice of priors and Markov chain Monte Carlo settings
☒	☐	For hierarchical and complex designs, identification of the appropriate level for tests and full reporting of outcomes
☒	☐	Estimates of effect sizes (e.g. Cohen's d, Pearson's r), indicating how they were calculated

Our web collection on statistics for biologists contains articles on many of the points above.

Software and code

Policy information about availability of computer code

Data collection	ELISA reader, CTL, Unicorn, other?
Data analysis	Graphpad PRISM (v9.2.0)

For manuscripts utilizing custom algorithms or software that are central to the research but not yet described in published literature, software must be made available to editors and reviewers. We strongly encourage code deposition in a community repository (e.g. GitHub). See the Nature Portfolio guidelines for submitting code & software for further information.

Data

Policy information about availability of data

All manuscripts must include a data availability statement. This statement should provide the following information, where applicable:

- Accession codes, unique identifiers, or web links for publicly available datasets
- A description of any restrictions on data availability
- For clinical datasets or third party data, please ensure that the statement adheres to our policy

All data is freely available upon request.

Field-specific reporting

Please select the one below that is the best fit for your research. If you are not sure, read the appropriate sections before making your selection.

☒ Life sciences ☐ Behavioural & social sciences ☐ Ecological, evolutionary & environmental sciences

For a reference copy of the document with all sections, see nature.com/documents/nr-reporting-summary-flat.pdf

Life sciences study design

All studies must disclose on these points even when the disclosure is negative.

Sample size	N=6 for mouse immunizations. This was expected to provide statistical significance for biologically relevant differences across vaccine approaches.
Data exclusions	No data was excluded.
Replication	antigen-specific serum antibodies from immunized mice were tested in two separate ELISAs. Neutralization experiments were conducted in three technical replicates and multiple hepatitis C culture viruses were tested to verify the presence of broadly neutralizing antibodies.
Randomization	Not relevant.
Blinding	Not relevant.

Reporting for specific materials, systems and methods

We require information from authors about some types of materials, experimental systems and methods used in many studies. Here, indicate whether each material, system or method listed is relevant to your study. If you are not sure if a list item applies to your research, read the appropriate section before selecting a response.

Materials & experimental systems

n/a	Involved in the study	
☐	☒	Antibodies
☐	☒	Eukaryotic cell lines
☒	☐	Palaeontology and archaeology
☐	☒	Animals and other organisms
☒	☐	Human research participants
☒	☐	Clinical data
☒	☐	Dual use research of concern

Methods

n/a	Involved in the study	
☒	☐	ChIP-seq
☒	☐	Flow cytometry
☒	☐	MRI-based neuroimaging

Antibodies

Antibodies used	Secondary goat anti-mouse IgG Horseradish peroxidase (Novex), Human anti-HCV antibodies AR1A, AR2A, AR3A, AR3B, AR3C, AR3D (Described and validated in DOI: 10.1038/nm1698), anti-HCV protein NS5A antibody 9E10 (described and validated in DOI: 10.1126/science.1114016)
Validation	Novex™ Goat anti-Mouse IgG (H+L) Cross-Adsorbed Secondary Antibody, HRP (product number: 15393896)<br>Antibodies AR1A, AR2A, AR3A, AR3B, AR3C, AR3D (Described and validated in DOI: 10.1038/nm1698)<br>antibody 9E10 (described and validated in DOI: 10.1126/science.1114016)

Eukaryotic cell lines

Policy information about cell lines

Cell line source(s)	S2 Drosophila Schneider cells
Authentication	*Describe the authentication procedures for each cell line used OR declare that none of the cell lines used were authenticated.*
Mycoplasma contamination	*Confirm that all cell lines tested negative for mycoplasma contamination OR describe the results of the testing for mycoplasma contamination OR declare that the cell lines were not tested for mycoplasma contamination.*
Commonly misidentified lines (See ICLAC register)	*Name any commonly misidentified cell lines used in the study and provide a rationale for their use.*

Animals and other organisms

Policy information about studies involving animals; ARRIVE guidelines recommended for reporting animal research

Laboratory animals	Female Balb/C mice
Wild animals	*Provide details on animals observed in or captured in the field; report species, sex and age where possible. Describe how animals were caught and transported and what happened to captive animals after the study (if killed, explain why and describe method; if released, say where and when) OR state that the study did not involve wild animals.*
Field-collected samples	*For laboratory work with field-collected samples, describe all relevant parameters such as housing, maintenance, temperature, photoperiod and end-of-experiment protocol OR state that the study did not involve samples collected from the field.*
Ethics oversight	All mouse studies included in this paper were planned and conducted according to the Federation of European Laboratory Animal Science Associations (FELASA) Guidelines and were approved by the Danish Animal Experiments Inspectorate (Approval number: 2013-15-2934-00902/BES).

Note that full information on the approval of the study protocol must also be provided in the manuscript.
